# Supplementary material for: AffyMAPSDetector: a software tool to characterize Affymetrix GeneChip™ expression arrays with respect to SNPs
Source: BMC Bioinformatics. 2007 Jul 30;8:276. doi: 10.1186/1471-2105-8-276 (PMC1959249; doi:10.1186/1471-2105-8-276)
Supplement: Additional file 13 — AffyMAPSDetector v1 source code. AffyMAPSDetector source code. [file 1471-2105-8-276-S13.zip › AffyMAPSDetector_v1_src/doc/affychip/snpdetector/model/package-tree.html]

affychip.snpdetector.model Class Hierarchy


|  |  |  |  |
| --- | --- | --- | --- |
| **Overview** \| **Package** \| Class \| Use \| **Tree** \| **Deprecated** \| **Index** \| **Help** | | |  |
| **PREV**   **NEXT** | **FRAMES**    **NO FRAMES**     **All Classes** |  |  |


---


## Hierarchy For Package affychip.snpdetector.model

**Package Hierarchies:**: All Packages

---

## Class Hierarchy

- class java.lang.Object
  - class affychip.snpdetector.model.**DataState** (implements java.io.Serializable)- class affychip.snpdetector.model.**GeneInfo**- class affychip.snpdetector.model.**Probe** (implements java.lang.Comparable)- class affychip.snpdetector.model.**ProbeSet** (implements java.lang.Comparable)- class affychip.snpdetector.model.**SnpDetector**
            - class affychip.snpdetector.model.**OptimizedSnpDetector**
            - class affychip.snpdetector.model.**SnpFinder**- class affychip.snpdetector.model.**SnpInfo**- class affychip.snpdetector.model.**SnpMatcher**- class java.lang.Thread (implements java.lang.Runnable)
                    - class affychip.snpdetector.model.**SnpMatcher.FileClosingShutdownHook**

---


|  |  |  |  |
| --- | --- | --- | --- |
| **Overview** \| **Package** \| Class \| Use \| **Tree** \| **Deprecated** \| **Index** \| **Help** | | |  |
| **PREV**   **NEXT** | **FRAMES**    **NO FRAMES**     **All Classes** |  |  |


---
